# Supplementary material for: HIVprotI: an integrated web based platform for prediction and design of HIV proteins inhibitors
Source: J Cheminform. 2018 Mar 9;10:12. doi: 10.1186/s13321-018-0266-y (PMC5845081; doi:10.1186/s13321-018-0266-y)
Supplement: Supplementary file 2 — Additional file 2. Source code of HIVProtI web server. [file 13321_2018_266_MOESM2_ESM.zip › HIVprotI_Source-code/hivprotI/weka-3-6-6/doc/weka/classifiers/trees/j48/ModelSelection.html]

ModelSelection


---


|  |  |  |
| --- | --- | --- |
| **Overview** \| **Package** \| **Class** \| **Tree** \| **Deprecated** \| **Index** \| **Help** \| **Weka's home** | |  |
| **PREV CLASS**   **NEXT CLASS** | **FRAMES**    **NO FRAMES**     **All Classes** |  |
| SUMMARY: NESTED \| FIELD \| CONSTR \| METHOD | DETAIL: FIELD \| CONSTR \| METHOD |  |


---


## weka.classifiers.trees.j48 Class ModelSelection

```
java.lang.Object
  weka.classifiers.trees.j48.ModelSelection
```

**All Implemented Interfaces:**: java.io.Serializable, RevisionHandler

**Direct Known Subclasses:**: BinC45ModelSelection, C45ModelSelection, NBTreeModelSelection, ResidualModelSelection

---

``` public abstract class ModelSelection extends java.lang.Object implements java.io.Serializable, RevisionHandler ```

Abstract class for model selection criteria.

**Version:**
:   $Revision: 1.8 $

**Author:**
:   Eibe Frank (eibe@cs.waikato.ac.nz)

**See Also:**: Serialized Form

---

**Constructor Summary**

`ModelSelection()`


| **Method Summary** | |
| --- | --- |
| `abstract  ClassifierSplitModel` | `selectModel(Instances data)`             Selects a model for the given dataset. |
| `ClassifierSplitModel` | `selectModel(Instances train, Instances test)`             Selects a model for the given train data using the given test data |

**Methods inherited from class java.lang.Object**

`equals, getClass, hashCode, notify, notifyAll, toString, wait, wait, wait`

**Methods inherited from interface weka.core.RevisionHandler**

`getRevision`

**Constructor Detail**

### ModelSelection

```
public ModelSelection()
```


**Method Detail**

### selectModel

```
public abstract ClassifierSplitModel selectModel(Instances data)
                                          throws java.lang.Exception
```

:   Selects a model for the given dataset.

    :   **Throws:**: `java.lang.Exception` - if model can't be selected

---


### selectModel

```
public ClassifierSplitModel selectModel(Instances train,
                                        Instances test)
                                 throws java.lang.Exception
```

:   Selects a model for the given train data using the given test data

    :   **Throws:**: `java.lang.Exception` - if model can't be selected


---


|  |  |  |
| --- | --- | --- |
| **Overview** \| **Package** \| **Class** \| **Tree** \| **Deprecated** \| **Index** \| **Help** \| **Weka's home** | |  |
| **PREV CLASS**   **NEXT CLASS** | **FRAMES**    **NO FRAMES**     **All Classes** |  |
| SUMMARY: NESTED \| FIELD \| CONSTR \| METHOD | DETAIL: FIELD \| CONSTR \| METHOD |  |


---
